# PD‐L2 Inhibits Protective Immunity, Th2 Cell Functional Quality, and GATA‐3 Expression During Filarial Nematode Infection

**DOI:** 10.1002/eji.70021

**Published:** 2025-08-04

**Authors:** Johanna A. Knipper, Sharon M. Campbell, Judith E. Allen, Matthew D. Taylor

**Affiliations:** ^1^ Institute of Immunology and Infection Research University of Edinburgh Edinburgh UK; ^2^ Max Planck Institute of Immunobiology and Epigenetics Freiburg Germany; ^3^ Lydia Becker Institute of Immunology and Inflammation School of Biological Sciences University of Manchester Manchester UK

**Keywords:** B cell, Helminth, immune‐regulation, T cell, Th2

## Abstract

Filarial nematodes infect over 200 million people, predominantly stimulating a Type 2 immune response. Protective immunity takes decades to become effective due to dominant immune suppression that develops during infection. Using *Litomosoides sigmodontis* infection as a murine model of filariasis, we previously demonstrated that PD‐1 co‐inhibition causes Th2 cells to become intrinsically dysfunctional or hypo‐responsive during infection, resulting in impaired protective immunity. Τhis study demonstrates that Th2 cell‐intrinsic hypo‐responsiveness is associated with a loss of GATA‐3 expression by CD4^+^ T cells from WT, but not PD‐L2^−/−^ mice. PD‐L2^−/−^ mice were more resistant to *L. sigmodontis* and had increased Th2 cell‐intrinsic functional quality. CD19^+^ B cells expressed PD‐L2, and Jh^−/−^ B cell‐deficient mice were more resistant to infection. However, Th2 cell‐intrinsic hypo‐responsiveness still developed in Jh^−/−^ mice, and restricting PD‐L2 deficiency to B cells using bone marrow chimaeras did not alter resistance to *L. sigmodontis* or Th2 cell‐intrinsic functional quality. Together, these data indicate that PD‐L2 inhibits protective immunity to *L. sigmodontis*, downregulates GATA‐3 in CD4^+^ T cells, and induces Th2 cell‐intrinsic hypo‐responsiveness. Whilst B cells play a suppressive role during infection, this is independent of PD‐L2 and B cells do not instigate Th2 cell‐intrinsic hypo‐responsiveness.

## Graphical abstract

During filarial nematode infection with *Litomosoides sigmodontis*, PD‐L2 down‐regulates Th2 cell expression of GATA‐3 and Th2 cytokines and enables parasite survival. Concurrently, B cells suppress protective immunity through a mechanism that does not involve PD‐L2.

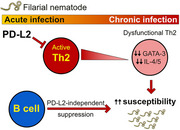

**Abbreviations**
d: day
FC: flow cytometry
Inf: infected
Mf: microfilaria
N: naïve
pi: postinfection
PleC: pleural cavity
tLN: thoracic lymph nodes
Treg: regulatory T cell
WT: wild‐type

## 1 Introduction

Helminths infect over 1 billion people worldwide [1], with filarial nematodes such as *Brugia malayi*, *Wuchereria bancrofti*, *Mansonella spp*., and *Onchocerca volvulus* accounting for approximately 200 million infected individuals [2, 3]. In common with the majority of helminths, filarial infections predominantly stimulate Type 2 immune responses in their host, although lesser Type 1 responses are also present [4]. Protective immunity takes decades to develop due to a combination of parasite‐induced immune suppression to aid parasite survival, and host‐induced immune downregulation to prevent immune pathology [5, 6]. Understanding the mechanisms by which Type 2 immune responses are regulated and suppressed is important for the development of vaccines against helminths and therapies to treat Type 2‐mediated diseases such as allergies.

A range of immunomodulatory mechanisms have been identified that extrinsically inhibit Th2 cell responses during chronic filariasis, including regulatory T cells (Tregs), macrophages, and B cells [4, 7, 8]. There is also evidence for Th2 cell‐intrinsic regulation during helminth infection, where Th2 cells become intrinsically dysfunctional or tolerised as infection progresses [8]. Helminth antigens are able to induce an anergic‐like phenotype in murine T cells [9, 10, 11], and anergic‐like T cell responses are seen in murine models of schistosomiasis and filariasis [12, 13, 14]. Human helminth infections are associated with impaired TCR signalling and expression of T cell anergy factors [15, 16, 17]. Filarial lymphedema in humans correlates with an exhausted phenotype in both CD4 and CD8 T cells [18, 19], and increased levels of exhausted CD4^+^ T cells are seen in chronic bovine *Fasciola hepatica* infection [20]. However, it is not known how T cell‐intrinsic dysfunction is induced, and whether these different examples of T cell‐intrinsic dysfunction represent common or distinct phenotypes.

Infection of permissive mouse strains with *Litomosoides sigmodontis* provides a valuable model to elucidate the immune regulatory pathways that inhibit Type 2 immunity, allowing filarial infections to establish chronic infections [7]. Using the *L. sigmodontis* infection model, we previously demonstrated that Th2 cells, despite remaining present at high levels, become intrinsically dysfunctional or hypo‐responsive during chronic infection and lose their ability to proliferate and produce Th2 cytokines in response to antigenic and mitogenic stimulation [12, 14, 21]. These hypo‐responsive Th2 cells are transcriptionally distinct from the active Th2 cells found during acute infection, but retain high levels of *IL‐4, IL‐5* and *IL‐13* mRNA, indicating that their inability to produce protein is controlled at the level of translation [12]. Blockade of the PD‐1 pathway during *L. sigmodontis* infection recovers Th2 cell‐intrinsic functionality and increases resistance [14], indicating that Th2 cell‐intrinsic hypo‐responsiveness is induced via the PD‐1 pathway. While PD‐1 co‐inhibition is linked to T cell exhaustion, *L. sigmodontis*‐elicited intrinsically hypo‐responsive Th2 cells have a gene expression profile distinct from exhausted T cells [12]. Instead, they upregulate the transcription factors c‐Maf and Egr2 that are associated with T cell anergy [12]. The hypo‐responsive Th2 cells also start producing IL‐21 protein, and neutralising the IL‐21R during infection increases resistance, indicating that they may play a regulatory role rather than being solely inert [12].

PD‐1 acts via PD‐L1 and/or PD‐L2 [22], and blockade of PD‐L2, but not PD‐L1, increases resistance to *L. sigmodontis* [14]. This suggests that, in contrast to PD‐L1‐mediated T cell exhaustion, PD‐L2 is inducing Th2‐cell intrinsic hypo‐responsiveness via PD‐1. In lymphatic filariasis patients, PD‐L2 polymorphisms are linked to disease, indicating PD‐L2 has a role in the human infection [23]. Thus, in this study, we employed PD‐L2^−/−^ mice to test whether PD‐L2 induces Th2‐cell intrinsic hypo‐responsiveness, and to identify through which cell type it is acting. PD‐L2^−/−^ mice showed increased resistance to *L. sigmodontis* and enhanced Th2 cell‐intrinsic functional quality, indicating that PD‐L2 inhibits protective immunity and instigates Th2 cell‐intrinsic hypo‐responsiveness. CD19^+^ B cells were found to express PD‐L2 during infection, and played an inhibitory role as B‐cell‐deficient Jh^−/−^ mice were more resistant to infection. However, Jh^−/−^ mice still developed Th2 cell‐intrinsic hypo‐responsiveness, and using bone marrow chimaeras to restrict PD‐L2 deficiency to B cells demonstrated that B cells do not suppress protective immunity via PD‐L2. Unexpectedly, CD4^+^ T cells from WT, but not PD‐L2^−/−^, mice were found to downregulate GATA‐3 protein during chronic infection. Thus, our data indicate that PD‐L2 and B cells inhibit protective immunity to *L. sigmodontis* via different mechanisms. PD‐L2 downregulates GATA‐3 in T cells and induces Th2 cell‐intrinsic hypo‐responsiveness, resulting in a dysfunctional Th2 cell response and susceptibility to infection.

## 2 Results

### 2.1 PD‐L2^−/−^ Mice Have Enhanced Type 2 Responses and Are More Resistant to *L. sigmodontis*

To determine whether PD‐L2 downregulates Type 2 immunity and inhibits resistance to *L. sigmodontis*, wild‐type (WT) and PD‐L2^−/−^ mice on the susceptible BALB/c background were infected with 30 *L. sigmodontis* L_3_ larvae, and parasite burden and immune responses were assessed at day (d) 20 and d 60 postinfection (pi). D 20 pi represents a time point when the Th2 cells are functionally active, and by d 60 pi, the Th2 cells at the infection site, the pleural cavity (PleC), have become intrinsically hypo‐responsive [12, 14]. *L. sigmodontis* infection in BALB/c mice becomes fully patent between d 55–60 pi with mature adults residing in the PleC, and transmission stage microfilaria (Mf) detectable in the blood [24, 25].

**FIGURE 1:**
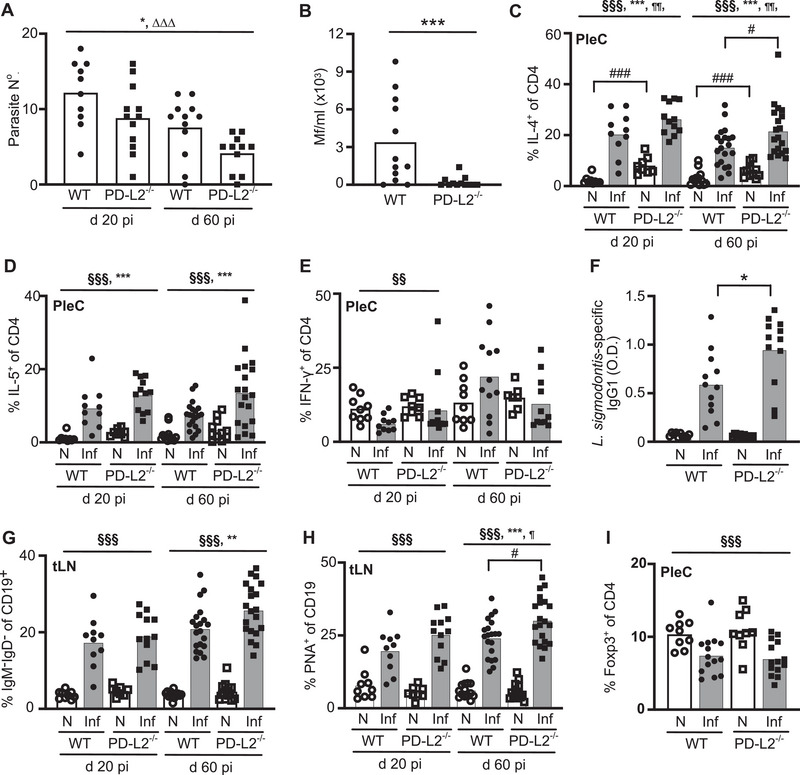
PD‐L2**^−^** ^/^ **^−^** mice have reduced parasite burdens and increased Type 2 immune responses. Parasite burdens and Type 2 immune responses were assessed in naïve (N, open symbols) and *L. sigmodontis*‐infected (Inf, closed symbols) WT (circles) and PD‐L2**^−^** ^/^ **^−^** (squares) mice at d 20 and d 60 pi. Symbols represent individual mice, and bars represent the mean. (A) Number of larval (d 20) or adult (d 60) parasites within the PleC. *Significant effect of genotype at *p* < 0.05, ^∆∆∆^ significant difference between timepoints at *p* < 0.001 (LM). (B) Number Mf/ml within the blood at d 60 pi. ***significant effect of genotype, *p* < 0.001 (GLM, negative binomial). (A, B) Data from two independent experiments; WT d20 *n* = 10, WT d60 *n* = 12, PD‐L2**^−^** ^/^ **^−^** d 20 *n* = 12, PD‐L2**^−^** ^/^ **^−^** d 60 *n* = 12. (C, D) Percentage of PleC CD4^+^ T cells producing IL‐4 (C) or IL‐5 (D). Data from 2 (d 20) or 3 (d60) independent experiments; naïve d 20 WT (*n* = 9) and PD‐L2**^−^** ^/^ **^−^** (*n* = 8), naïve d 60 WT (*n* = 13) and PD‐L2**^−^** ^/^ **^−^** (*n* = 11), infected d 20 WT (*n* = 10) and PD‐L2**^−^** ^/^ **^−^** (*n* = 12), infected d 60 WT (*n* = 20) and PD‐L2**^−^** ^/^ **^−^** (*n* = 19). (E) Percentage of PleC CD4^+^ T cells producing IFN‐γ. Data from two independent experiments; naïve d 20 WT (*n* = 9) and PD‐L2**^−^** ^/^ **^−^** (*n* = 8), naïve d 60 WT (*n* = 9) and PD‐L2**^−^** ^/^ **^−^** (*n* = 7), infected d 20 WT (*n* = 10) and PD‐L2**^−^** ^/^ **^−^** (*n* = 12), infected d 60 WT (*n* = 12) and PD‐L2**^−^** ^/^ **^−^** (*n* = 11). (F) Levels of *L*. *sigmodontis* Ag‐specific IgG1 within the blood at d 60 pi. Data from two independent experiments; naïve WT (*n* = 10) and PD‐L2**^−^** ^/^ **^−^** (*n* = 10), infected WT (*n* = 12) and PD‐L2**^−^** ^/^ **^−^** (*n* = 11). (G & H) Percentage of class‐switched IgM**^−^** IgD**^−^** CD19^+^ B cells (G) and PNA^+^ germinal centre B cells (H) within the tLN. Data from 2 (d 20) or 3 (d 60) independent experiments; N WT (d20 *n* = 9, d 60 *n* = 14), infected WT (d20 *n* = 10, d 60 *n* = 20), N PD‐L2**^−^** ^/^ **^−^** (d20 *n* = 8, d 60 *n* = 14), infected PD‐L2**^−^** ^/^ **^−^** (d20 *n* = 12, d60 *n* = 19‐20). (I) Percentage of PleC CD4^+^ T cells expressing Foxp3 at d 60 pi. Data from 2 independent experiments; N WT (*n* = 9), infected WT (*n* = 14), N PD‐L2**^−^** ^/^ **^−^** (*n* = 9), infected PD‐L2**^−^** ^/^ **^−^** (*n* = 13). (C–I) Significant effect of infection at ^§§§^ *p* < 0.001 or ^§§^ *p* < 0.01, genotype at ***p < 0.001 or *p < 0.05, infection*genotype at ^¶¶^ *p* < 0.01 or ^¶^ *p* < 0.05 (LM). ###*p* < 0.001, #*p* < 0.05 (Tukey's HSD).

PD‐L2^−/−^ mice had a significant 28% reduction in the numbers of L_4_ larvae within the PleC by d 20 pi, and a 41% reduction in the numbers of adult parasites at d 60 pi (Figure 1A). The decrease in adult parasites at d 60 pi was associated with significantly reduced levels of Mf in the blood of PD‐L2^−/−^ mice compared with WT mice (Figure 1B). Thus, PD‐L2‐deficient mice had significantly increased resistance to *L. sigmodontis* compared with WT mice.

To determine whether PD‐L2 deficiency resulted in enhanced Th2 responses, we measured CD4^+^ T cell responses within the PleC using flow cytometry (FC) (Figure S1A). PD‐L2^−/−^ mice had significantly reduced total numbers of PleC cells at d 20 pi, but not at d60 pi (Figure S2A), and there was a significant reduction in total CD4^+^ T cell numbers in the PleC of both naïve and infected PD‐L2ko mice at both d 20 and 60 pi (Figure S2B). In contrast to the decreased CD4^+^ T cell numbers, naïve and infected PD‐L2^−/−^ mice had a significantly higher proportion of PleC CD4^+^ T cells producing IL‐4 and IL‐5 at both d 20 and 60 pi (Figure 1C,D; Figure S1B,C). PD‐L2 deficiency had no effect on the production of IFN‐γ by PleC CD4^+^ T cells (Figure 1E; Figure S1D). Thus, PD‐L2‐deficient mice mount a stronger Th2 response towards *L. sigmodontis*.

Alongside increased Th2 cytokine responses, infected PD‐L2^−/−^ mice had significantly elevated levels of *L. sigmodontis*‐specific IgG1 within their serum (Figure 1F). Although PD‐L2 deficiency did not alter the total cell number, or total number of CD19^+^ B cells, within the LN draining the PleC (tLN) of naïve, d 20, or d 60 infected mice (Figures S2C,D and S3A), there were significantly increased proportions of IgM^−^IgD^−^ class‐switched and PNA^+^ germinal center CD19^+^ B cells within the tLN of PD‐L2^−/−^ mice compared with WT mice at d 60 pi (Figure 1G,H; Figure S3B,C). This increased humoral response was not associated with a change in the proportion of tLN CXCR5^+^ Tfh cells (Figures S2E and S3D) or tLN CD138^+^ plasma B cells (Figures S2F and S3E) in PD‐L2^−/−^ mice compared with WT. Whilst Foxp3^+^ Tregs inhibit protective immunity and Th2 responses to *L. sigmodontis* [21, 26, 27], the enhanced resistance and Th2 responses in PD‐L2^−/−^ mice did not associate with changes in the frequency of PleC Foxp3^+^ T cells [Figure 1I; Figure S1E]. Thus, PD‐L2^−/−^ mice have enhanced resistance, Th2 responses, and humoral responses to *L. sigmodontis*.

### 2.2 PD‐L2 Inhibits Th2 Cell‐intrinsic Functional Quality, and *L. sigmodontis* Infection Downregulates Th2 Cell GATA‐3 in a PD‐L2 Dependent Manner

**FIGURE 2:**
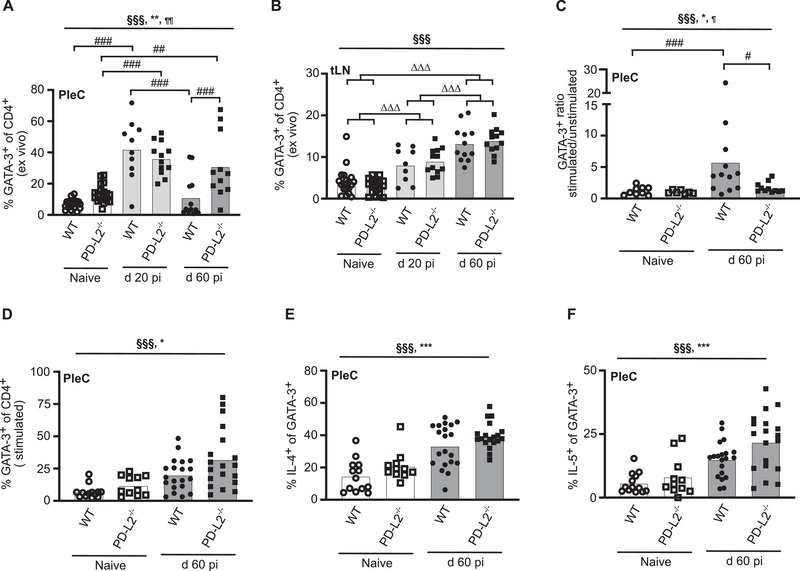
PD‐L2^−/−^ mice have increased GATA‐3 and Th2 cell functional quality. GATA‐3 expression, and production of IL‐4 and IL‐5 by CD4^+^GATA‐3^+^ Th2 cells were assessed by FC in naïve (N, open symbols) and *L. sigmodontis*‐infected (Inf, closed symbols) WT (circles) and PD‐L2^−/−^ (squares) mice at d 20 and 60 pi. Naïve groups were pooled across timepoints. Symbols represent individual mice, and bars represent the mean. (A, B) Percentage of unstimulated PleC (A) and tLN (B) CD4^+^ T cells expressing GATA‐3. Data from 2 independent experiments; N WT (*n* = 19), N PD‐L2^−/−^ (*n* = 18), infected WT (d20 *n* = 9–10, d 60 *n* = 12), infected PD‐L2^−/−^ (d20 *n* = 12, d60 *n* = 11–12). Naïve animals were pooled across timepoints, and data were analysed using LM. (C) Ratio of stimulated to unstimulated PleC CD4^+^ T cells expressing GATA‐3 at d 60 pi. Data from two independent experiments; N WT (*n* = 9), N PD‐L2^−/−^ (*n* = 7), infected WT (*n* = 12), infected PD‐L2^−/−^ (*n* = 11). (D) Percentage of stimulated PleC CD4^+^ T cells expressing GATA‐3 at d 60 pi. Data from three independent experiments; N WT (*n* = 13), N PD‐L2^−/−^ (*n* = 11), infected WT (*n* = 20), infected PD‐L2^−/−^ (*n* = 19). (E, F) Percentage of PleC CD4^+^GATA‐3^+^ T cells producing IL‐4 (E) and IL‐5 (F) at d 60 pi. Data from three independent experiments; N WT (*n* = 13), N PD‐L2^−/−^ (*n* = 11), infected WT (*n* = 20), infected PD‐L2^−/−^ (*n* = 19). (A–F) Significant effect of infection at ^§§§^ *p* < 0.001, genotype at **p < 0.01 or *p < 0.05, and infection*genotype at ^¶¶¶^ *p* < 0.001 or ^¶^ *p* < 0.05 (LM). ^#^ *p* < 0.05, ^##^ *p* < 0.01, ^###^ *p* < 0.001 (Tukey's HSD), **^∆∆∆^** significant difference between time points independent of genotype, *p* < 0.001 (Tukey's HSD).

To determine whether PD‐L2 induces Th2 cell‐intrinsic hypo‐responsiveness, the functional quality of Th2 cells was assessed during infection of WT and PD‐L2^−/−^ mice. GATA‐3, a key Th2 transcription factor, was used to identify CD4^+^GATA‐3^+^ Th2 cells. Initially, GATA‐3 expression by CD4^+^ T cells was assessed directly ex vivo in the absence of stimulation. Infected WT mice showed a significant increase in the proportion of PleC CD4^+^GATA‐3^+^ T cells at d20 pi compared with naïve controls, consistent with the development of an early functional Th2 response (Figure 2A; Figure S4A). Unexpectedly, at d60 pi, there was a pronounced significant decrease in the proportion and total numbers of WT PleC CD4^+^ T cells expressing GATA‐3 (Figure 2A; Figure S2G). Thus, at d 60 pi, naïve and infected WT mice had equivalent proportions of PleC CD4^+^ T cells expressing GATA‐3, despite previous studies showing 20–70% of CD4^+^ T cells in the PleC are IL‐4gfp^+^ Th2 cells and express *GATA‐3* mRNA at this time point [12, 14]. Th2 cell‐intrinsic hypo‐responsiveness is restricted to the PleC and not observed in the tLN [14]. TLN CD4^+^ T cells retained GATA‐3 protein expression at d 60 pi (Figure 2B), indicating that the loss of GATA‐3 is restricted to the hypo‐responsive phenotype at the infection site. In contrast to WT mice, there was no downregulation in the proportion or total numbers of PleC CD4^+^ T cells expressing GATA‐3 protein in the PD‐L2^−/−^ mice between d 20 and 60 pi (Figure 2A; Figure S2G), and WT mice had significantly lower proportion of CD4^+^ T cells expressing GATA‐3 than PD‐L2^−/−^ mice (Figure 2A). Thus, the hypo‐responsive Th2 cell phenotype at d 60 pi associates with a PD‐L2‐dependent loss of GATA‐3 protein expression in CD4^+^ T cells.

Th2 cell intrinsic hypo‐responsiveness is functionally defined by the loss of antigen‐specific proliferation and cytokine production, but can be visualised as a reduced proportion of Th2 cells actively producing Th2 cytokines following intracellular cytokine staining [14, 21]. Thus, intracellular cytokine staining was used to determine whether the proportion of CD4^+^GATA‐3^+^ Th2 cells actively producing IL‐4 and IL‐5 was increased in PD‐L2^−/−^ mice. Stimulating the d 60 PleC CD4^+^ T cells with PMA and ionomycin was found to partially restore GATA‐3 protein expression by CD4^+^ T cells from infected WT mice. Stimulation of CD4^+^ T cells from naïve WT and PD‐L2^−/−^ naïve mice, and infected PD‐L2^−/−^ mice, resulted in equivalent small fold‐increases (1–1.7‐fold) compared with ex vivo GATA‐3 expression of unstimulated CD4 T cells (Figure 2C; Figure S4B). In contrast, CD4^+^ T cells from d 60 infected WT mice showed a significantly higher 5.8‐fold increase in GATA‐3 expression following stimulation with PMA and ionomycin (Figure 2C). Thus, stimulation of d 60 CD4^+^ T cells from infected WT mice recovered GATA‐3 expression towards the levels seen in PD‐L2^−/−^ mice ex vivo without stimulation (Figure 2A,D).

Although CD4^+^ T cells from WT mice have downregulated GATA‐3 protein at d 60 pi, their subsequent upregulation of GATA‐3 protein in response to PMA and ionomycin stimulation meant that GATA‐3 could still be used to quantify the proportion of Th2 cells actively producing cytokines in both WT and PD‐L2^−/‐^ mice. Thus, the proportion of CD4^+^GATA‐3^+^ T cells producing IL‐4 and IL‐5 was measured to test whether PD‐L2 deficiency prevents Th2 cell‐intrinsic hypo‐responsiveness. A significantly higher proportion of CD4^+^GATA‐3^+^ Th2 cells from both naïve and infected PD‐L2^−/−^ mice produced IL‐4 and IL‐5 cytokines (Figure 2E,F; Figure S4C, D), indicating that CD4^+^GATA3^+^ Th2 cells from PD‐L2ko mice have a higher intrinsic functional quality. Together, these data suggest that PD‐1 is acting via PD‐L2 to induce Th2 cell intrinsic hypo‐responsiveness, and that PD‐L2‐dependent downregulation of GATA‐3 is part of the hypo‐responsive phenotype.

### 2.3 B Cells Protect Against Larval Establishment, but Inhibit Protective Immunity During Chronic Infection

**FIGURE 3:**
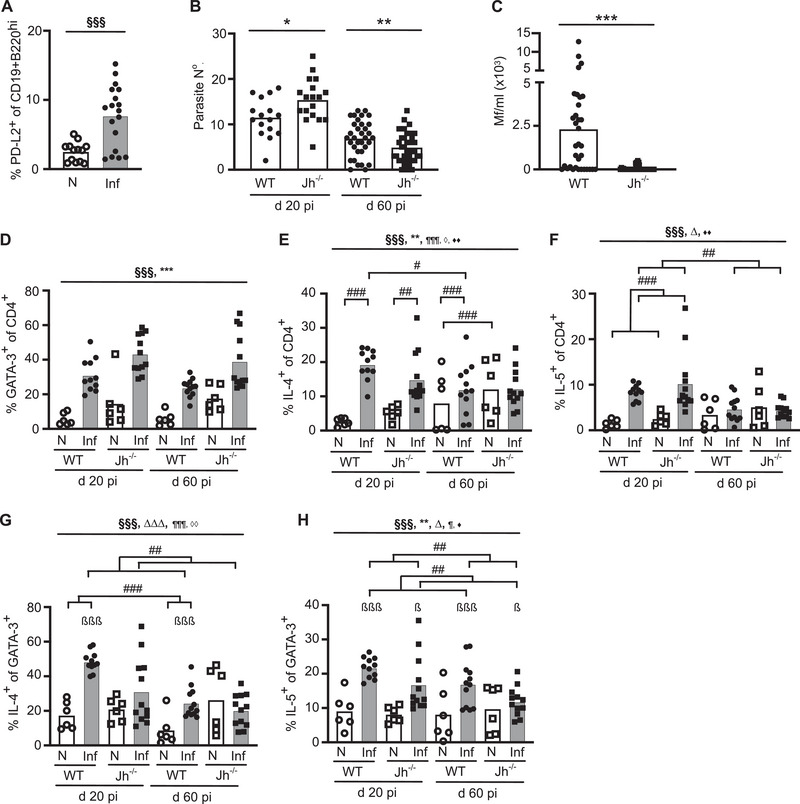
B cell‐deficient mice are more resistant to infection but still develop Th2 cell‐intrinsic hypo‐responsiveness. Symbols represent individual mice, and bars represent the mean. Open and closed symbols represent naïve and infected mice, respectively. (A) Percentage of tLN CD19^+^ B cells from naïve and d 60 *L. sigmodontis*‐infected WT mice expressing PD‐L2. Data from three independent experiments: N (*n* = 13), Inf (*n* = 18), and analysed via LM. (B–H) Parasite burdens and Type 2 immune responses were assessed in naïve (N) and *L. sigmodontis*‐infected (Inf) WT (circles) and Jh^−/−^ (squares) mice at d 20 and 60 pi. (B) Number of larval (d20) or adult (d60) parasites within the PleC. (C) Number Mf/ml within the blood at d 60 pi. (B, C) Data representative of four independent experiments: WT (d20 *n* = 16, d60 *n* = 34), Inf (d20 *n* = 18, d60 *n* = 37), and analysed using a LM (B) or GLM with negative binomial (C). (D) Percentage of stimulated PleC CD4^+^ T cells expressing GATA‐3. (E, F) Percentage of PleC CD4^+^ T cells producing IL‐4 (E) and IL‐5 (F). (G, H) Percentage of PleC CD4^+^GATA‐3^+^ T cells producing IL‐4 (G) and IL‐5 (H). (D–H) Data from two independent experiments: N WT & Jh^−/−^ (d20 *n* = 6, d 60 *n* = 6), Inf WT (d20 *n* = 11, d60 *n* = 12), Inf Jh^−/−^ (d20 *n* = 12, d60 *n* = 12). (A–H) Significant effect of infection at ^§§§^ *p* < 0.001, effect of genotype at *p < 0.05 or **p < 0.01 or ***p < 0.001, difference between timepoints at ^∆^ *p* < 0.05 or ^∆∆∆^ *p* < 0.001, effect of infection*genotype at ^¶¶¶^ *p* < 0.001, effect of timepoint*genotype at ^◊^ *p* < 0.05 or ^◊◊^ *p* < 0.01, effect of timepoint*infection at ^♦♦^ *p* < 0.01 (LM). Significant difference between infected and naïve at ^β^ *p* < 0.05 or ^βββ^ *p* < 0.001 (Tukey's HSD). ^#^ *p* < 0.05, ^##^ *p* < 0.01, ^###^ *p* < 0.001 (Tukey's HSD).

As B cells express PD‐L2 [22], and *L. sigmodontis*‐infected µMT B cell‐deficient mice develop lower microfilaremia [28], we investigated whether B cells induce Th2 cell‐intrinsic hypo‐responsiveness via PD‐L2. Flow cytometric analysis demonstrated distinct populations of CD19^hi^B220^lo^ and CD19^lo^B220^hi^ B cells expressing PD‐L2 in the PleC of naïve WT mice, and this population remained constant during *L. sigmodontis* infection (Figure S5A). Within the tLN, PD‐L2 was significantly upregulated on CD19^+^ B cells at d 60 pi compared with naïve mice (Figure 3A; Figure S5B). Thus, PleC B cells express PD‐L2 constitutively, and tLN B cells upregulate PD‐L2 during *L. sigmodontis* infection.

To test the role of B cells in resistance to *L. sigmodontis*, Jh^−/−^ B cell‐deficient mice on the susceptible BALB/c background were infected with *L. sigmodontis* and parasite burdens were measured. Initially, Jh^−/−^ mice were more susceptible than WT mice, harbouring a significantly increased number of L_4_ larvae at d 20 pi (Figure 3B). However, by d 60 pi, Jh^−/−^ mice had significantly lower numbers of adult parasites and blood‐circulating Mf than infected WT mice (Figure 3B,C). This suggests that B cells play stage‐dependent roles, being protective during the initial larval infection stages, but switching to an inhibitory role during chronic infection.

To determine whether B cells inhibit protective immunity by inducing Th2 cell‐intrinsic hypo‐responsiveness, CD4^+^ Th2 cell responses of *L. sigmodontis*‐infected Jh^−/−^ mice were assessed. B cell deficiency had no effect on the total numbers of PleC cells in infected mice at d 20 or d 60 pi, although there was a significant reduction in the total numbers of PleC CD4^+^ T cells in Jh^−/−^ mice at d 60 pi (Figure S2H,I). As these experiments were performed prior to discovering the GATA‐3 downregulation, CD4^+^ T cell expression of GATA‐3 was only assessed following PMA and ionomycin stimulation. Following stimulation with PMA and ionomycin, both naïve and infected Jh^−/−^ mice had a significantly higher proportion of CD4^+^ T cells expressing GATA‐3 (Figure 3D), suggesting that B cells negatively regulate Th2 cell expansion. The increase in Th2 cells did not correlate with a change in the proportions of Foxp3^+^ Tregs (Figure S2J).

Consistent with the development of Th2 cell‐intrinsic hypo‐responsiveness, the percentage of CD4^+^ T cells and CD4^+^GATA‐3^+^ Th2 cells producing IL‐4 and IL‐5 significantly decreased between d 20 and 60 pi in infected WT mice (Figure 3E–H). CD4^+^ T cells from Jh^−/−^ mice showed a similar pattern, with the proportion of CD4^+^ T cells producing IL‐4 and IL‐5 remaining equivalent to the WT (Figure 3E–H). However, in comparison to WT, a significantly lower proportion of CD4^+^GATA‐3^+^ T cells from infected Jh^−/−^ mice produced IL‐4 and IL‐5, indicating that Th2 cell‐intrinsic functional quality was lower in the absence of B cells (Figure 3G,H). Together, this suggests that whilst B cells negatively regulate the proportion of CD4^+^GATA‐3^+^ Th2 cells, they are not required for the development of Th2 cell‐intrinsic hypo‐responsiveness, and rather they enhance Th2 cell‐intrinsic functional quality.

### 2.4 B Cell‐derived PD‐L2 Does Not Affect Resistance or Instigate Th2 Cell Hypo‐responsiveness

Infections of Jh^−/−^ mice indicate that B cells both promote and inhibit immune responses towards *L. sigmodontis*. Thus, whilst the data from B cell‐deficient mice indicate that B cells enhance rather than inhibit Th2 cell‐intrinsic functional quality, this interpretation may be confounded by their dual roles. Also, it is not known whether their inhibitory role is dependent upon PD‐L2. To focus on their inhibitory role, and to determine whether B cells regulate protective immunity and Th2 cell intrinsic hypo‐responsiveness via PD‐L2, bone marrow chimaeras were created to specifically restrict PD‐L2 deficiency to B cells by lethally irradiating Jh^−/−^ mice and reconstituting them with 80% Jh^−/−^ bone marrow supplemented with 20% PD‐L2^−/−^ bone marrow. Thus, B cells can only derive from PD‐L2^−/−^ bone marrow, whilst all other cells can derive from PD‐L2 sufficient WT bone marrow. In this model, a proportion of non‐B cells can also derive from PD‐L2^−/−^, rather than WT, bone marrow cells. To control for this, a group of irradiated Jh^−/−^ mice were reconstituted with 80% WT bone marrow and 20% PD‐L2^−/−^ bone marrow. As a WT control, in which all cells are PD‐L2 sufficient, irradiated Jh^−/−^ mice were reconstituted with 80% Jh^−/−^ bone marrow and 20% WT bone marrow.

**FIGURE 4:**
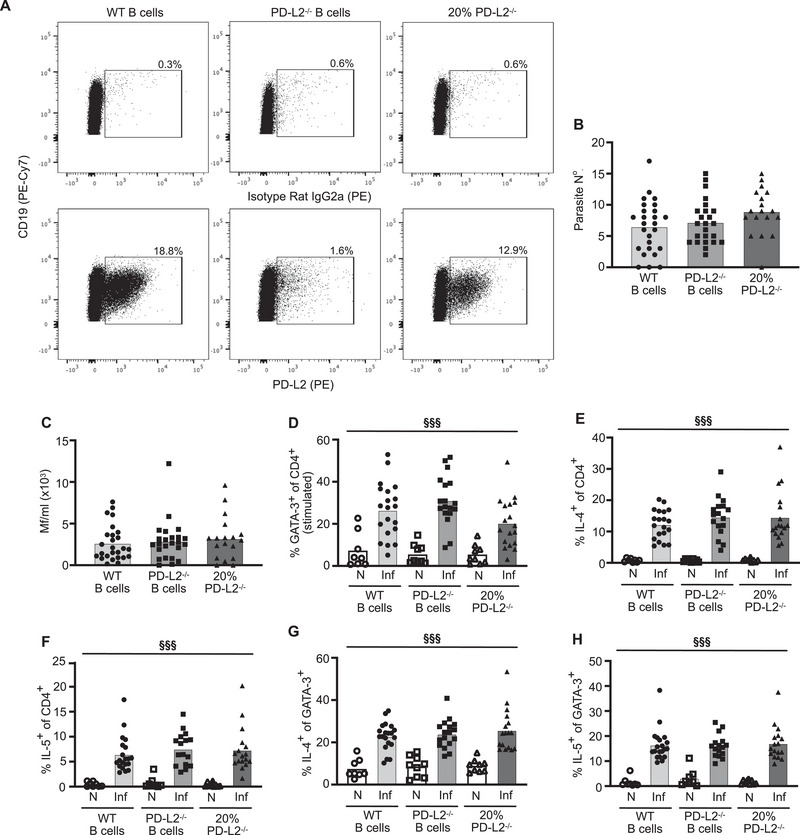
B cells inhibit protective immunity independently of PD‐L2. Parasite burdens and Th2 responses were assessed in naïve (N, open symbols) and d 60 *L. sigmodontis*‐infected (Inf, closed symbols) B cell chimaeras that contained B cells deficient for PD‐L2 (squares), or control chimaeras generated with WT B cells (circles) or 80%/20% WT/PD‐L2^−/−^ (triangles) bone marrow. (A) Representative flow staining showing expression of PD‐L2 on CD19^+^ B cells. (B, C) Number of adult parasites within the PleC (B), and number of Mf/mL within the blood (C). Data from 3 independent experiments: WT (*n* = 26), B cell PD‐L2^−/−^ (*n* = 24), 20% PD‐L2^−/−^ (*n* = 18). (D) Percentage of stimulated PleC CD4^+^ T cells expressing GATA‐3. (E, F) Percentage of PleC CD4^+^ T cells producing IL‐4 (E) and IL‐5 (F). (G, H) Percentage of PleC CD4^+^GATA‐3^+^ T cells producing IL‐4 (G) and IL‐5 (H). (D–G) Data from two independent experiments: N WT B cell (*n* = 8), Inf WT B cell (*n* = 20), N B cell PD‐L2^−/−^ (*n* = 8–9), Inf B cell PD‐L2^−/−^ (*n* = 16‐18), N 20% PD‐L2^−/−^ (*n* = 9), Inf 20% PD‐L2^−/−^ (*n* = 17–18). (B–G) ^§§§^significant effect of infection at *p* < 0.001 (LM).

As there is a clearly defined population of CD19^+^PD‐L2^+^ B cells in the PleC (Figure S5A), FC of PleC CD19^+^ B cells was performed to verify PD‐L2 deficiency. The PleC PD‐L2^+^ B cell population was absent in chimaeras in which PD‐L2 deficiency was restricted to B cells (Figure 4A). FC was used to determine whether B‐cell‐restricted PD‐L2 deficiency affected the main T‐ and B‐cell populations. Independent of infection status, there were no differences between the three groups in the total numbers of PleC or tLN cells (Figure S6A,B), PleC or tLN CD4^+^ T cells (Figure S6C,D), PleC Foxp3^+^ Tregs (Figure S6E), or tLN CXCR5^+^ Tfh cells (Figure S6F). There were also no differences in total numbers of tLN CD19^+^ B cells (Figure S6G), or the proportions of PNA^+^ germinal centre B cells, IgM^−^IgD^−^ class‐switched B cells, or CD138^+^ plasma cells, except for a slightly increased baseline of CD138^+^ plasma cells in the naive 20% PD‐L2^−/−^ control group (Figure S6H–J). Thus, except for the expression of PD‐L2 by B cells, the three groups were equivalent in their main T‐ and B‐cell populations.

At d 60 pi, B cell‐restricted PD‐L2^−/−^ chimaeras had equivalent numbers of adult parasites and levels of Mf within their blood to the control groups (Figure 4B,C), indicating that B cells are not inhibiting protective immunity via PD‐L2. The absence of PD‐L2 on B cells also had no effect on PleC Th2 responses. The proportion of d 60 CD4^+^ T cells expressing GATA‐3 following PMA and ionomycin stimulation remained the same across the three groups (Figure 4D). Similarly, the proportions of CD4^+^ and CD4^+^GATA‐3^+^ T cells producing IL‐4 and IL‐5 were equivalent across the three groups (Figure 4E–H). Thus, whilst B cells suppress protective immunity to *L. sigmodontis* (Figure 3B,C), this is independent of PD‐L2, and B cell expression of PD does not contribute to the development of Th2 cell‐intrinsic hypo‐responsiveness.

## 3 Discussion

Th2 cell‐intrinsic regulation is an important way of controlling Type 2 immune responses during helminth infections, impacting both resistance and pathology [9, 10, 11, 12, 13, 14, 15, 16, 17, 18, 19, 20]. However, the underlying mechanisms by which Th2 cells are intrinsically downregulated are not well defined. In this study, we demonstrate that Th2 cell‐intrinsic hypo‐responsiveness associates with a PD‐L2‐dependent loss of GATA‐3 expression by T cells at the infection site. PD‐L2 induced Th2 cell‐intrinsic hypo‐responsiveness in a B cell‐independent manner, with PD‐L2 deficient mice showing increased resistance and enhanced Th2 cell‐intrinsic functional quality. This indicates that the PD‐1–PD‐L2 pathway plays an important role in negatively regulating Th2 cell‐intrinsic functional quality and GATA‐3 expression during chronic infection.

The downregulation of GATA‐3 protein in the hypo‐responsive PleC Th2 cell population suggests that their intrinsically dysfunctional phenotype is linked to loss of this key Th2 transcription factor [29]. The decrease in GATA‐3 expression is unlikely to be due to a loss of Th2 cells, as 20–60% of the PleC CD4^+^ T cells remain IL‐4gfp^+^ Th2 cells during the hypo‐responsive phase of infection [14]. The intrinsically hypo‐responsive Th2 cell population also retains high levels of mRNA for *IL‐4*, *IL‐5*, *IL‐13*, and *GATA‐3*, indicating that they maintain a Th2 phenotype at the mRNA level despite their impaired ability to produce Th2‐related proteins [12]. As production of IL‐4 protein can be controlled at the level of translation [30], the impaired ability to produce Th2 cytokine and GATA‐3 protein may be due to translational regulation. Stimulation with PMA and ionomycin was sufficient to recover expression of GATA‐3 protein, indicating that a sufficiently strong T cell stimulation can rapidly overcome the GATA‐3 translational block. Whilst stimulation was not sufficient to recover protein production for Th2 cytokines [14], this may be because GATA‐3 is required for Th2 cytokine production of Th2 cytokines and a 4 h stimulation is not enough time for the restored GATA‐3 expression to turn on cytokine production.

The intrinsically hypo‐responsive Th2 cells found during chronic *L. sigmodontis* infection have a distinct gene expression profile from active Th2 cells found during acute infection with *L. sigmodontis* and *Nippostrongylus brasiliensis* [12]. Whilst this change mostly represents a switch from a classical Th2 phenotype to a tolerised or inert Th2 phenotype, a proportion of the Th2 cells develop a hybrid Th1/Th2 phenotype [12]. A subset of the Th2 cells also starts producing IL‐21, which inhibits protective immunity to *L. sigmodontis*, suggesting the development of a regulatory population [12]. This indicates that the Th2 cell phenotype becomes plastic as *L. sigmodontis* infection progresses, allowing the Th2 cells to diverge towards alternative functional phenotypes. As GATA‐3 is required to initiate and maintain the Th2 cell phenotype [29], its loss between d 20 and d 60 of infection is a potential mechanism by which Th2 cells are able to diverge from a classical Th2 phenotype. GATA‐3 downregulation was PD‐L2 dependent, suggesting that the PD‐1–PD‐L2 pathway directly or indirectly controls GATA‐3 expression during chronic Type 2 responses. In turn, this suggests that the PD‐1–PD‐L2 pathway may act as a plasticity factor that determines the stability of a Th2 response during chronicity.

Our data indicate that B cells play stage‐dependent roles during *L. sigmodontis* infection. During the initial infection stages, B‐cell‐deficient mice had higher parasite burdens, indicating an early protective role against the larvae. There is an initial large attrition of invading larvae within the first week of infection, with only 30% of larvae surviving [25, 31]. B1 B cells and secreted IgM play roles in killing *Brugia* larvae within the first 2 weeks of infection [32, 33], and *L. sigmodontis* infection results in the formation of fat‐associated lymphoid clusters within the PleC that support rapid innate‐like B cell responses [34]. Thus, in primary infections, the production of natural antibodies by B cells may be involved in the initial larval killing. However, during the chronic adult stage, B cell‐deficient mice had lower parasite burdens, indicating a switch from a protective to suppressive role as infection progressed. Following the initial larval killing in WT mice, parasite burdens remain stable until d 70 pi [25], and our data indicate that immune inhibition by B cells is one reason why the host is unable to kill *L. sigmodontis* during this period. Although suppression by B cells often involves IL‐10, neutralising the IL‐10R during chronic *L. sigmodontis* infection does not affect susceptibility [21]; thus, IL‐10 is unlikely to be the B cells’ dominant mechanism of suppression.

Restricting PD‐L2 deficiency to B cells did not affect their inhibitory role, and B cell‐derived PD‐L2 was not involved in the development of Th2 cell‐intrinsic hypo‐responsiveness. In fact, B cells appeared to enhance rather than inhibit Th2 cell functional quality, as the proportion of Th2 cells producing IL‐4 and IL‐5 in B cell‐deficient mice decreased. Thus, the PD‐L2 expressing cell type responsible for inhibiting protective immunity, and downregulating GATA‐3 in Th2 cells and driving them towards a dysfunctional phenotype is still unknown. IL‐4 drives PD‐L2 expression on macrophages [35, 36], and PD‐L2 is expressed by alternatively active macrophages (AAM) during *L. sigmodontis* infection [37, 38]. Whilst suppression of T cell proliferation in vitro by *L. sigmodontis*‐elicited AAM is PD‐L2 independent [14], in other contexts, IL‐4‐elicited AAM do suppress in vitro T cell proliferation via PD‐L2 [36, 39]. In vivo, blocking monocyte recruitment to the PleC during *L. sigmodontis* infection of susceptible mice increased resistance and Th2 cell‐intrinsic functional quality, suggesting a role for monocytes in initiating Th2 cell‐intrinsic hypo‐responsiveness [37]. Both susceptible and resistant mouse strains show recruitment of monocytes to the PleC during infection, and in resistant C57BL/6 mice, these monocytes rapidly transition into a resident macrophage phenotype that does not express PD‐L2. However, in susceptible BALB/c mice, this transition is blocked, resulting in the accumulation of a PD‐L2^+^ ‘converting’ macrophage population [7, 37, 40]. Th2 cells are required for monocyte transition into the resident macrophage niche in resistant *L. sigmodontis*‐infected mice, and during *S. mansoni* infection [40, 41]. These PD‐L2^+^ monocyte‐derived cells are very abundant in the PleC of *L. sigmodontis*‐infected BALB/c mice [40] and thus a likely cell to interact with local T cells. This would suggest a negative feedback loop in which impaired Th2 responses in susceptible BALB/c mice, potentially caused by the early dominant Foxp3^+^ Treg responses to *L. sigmodontis* [21, 26], lead to the accumulation of PD‐L2^+^ monocyte‐derived macrophages in the PleC of susceptible BALB/c mice. The monocytes then further inhibit the Th2 cells by downregulating GATA‐3 and instigating Th2 cell‐intrinsic hypo‐responsiveness.

Th2 cell intrinsic regulation is used to control Type 2 immune responses in a range of helminth infection settings. A better understanding is needed of the mechanisms by which Th2 cells are intrinsically regulated, and whether Th2 cell‐intrinsic regulation is important in other Type 2 settings, such as allergic inflammation. This knowledge will help develop vaccines and treatments for helminths that can counter Th2‐cell intrinsic regulation. It also has the potential to be used to treat Type 2‐mediated diseases by turning off the underlying pathogenic Th2 cells.

## 4 Data Limitations and Perspectives

Whilst the hypo‐responsive phenotype has previously been shown to result in an antigen‐specific loss of proliferation and cytokine production [14, 21], measuring antigen‐specific PleC T cell responses requires pooling cells from multiple mice. This has ethical considerations in terms of mouse numbers, and pooled samples prevent readouts from individual mice. Determining the proportion of Th2 cells producing cytokine in response to PMA/ionomycin stimulation was used to visualise the hypo‐responsive phenotype in this study, as it is a reliable surrogate readout for antigen‐specific responses [12, 14], requires three‐ to fourfold fewer mice, and provides readouts from individual mice. However, without antigen‐specific stimulation, it is not possible to determine whether the increased proportion of CD4^+^GATA‐3^+^ T cells producing Th2 cytokines following PMA/ionomycin stimulation equates to an increase in vivo levels of IL‐4 and IL‐5.

A question this data cannot answer is whether PD‐L2 is acting alone or alongside other factors to instigate Th2 cell‐intrinsic hypo‐responsiveness. A comparison of IL‐4 and IL‐5 production by CD4^+^GATA‐3^+^ T cells from PD‐L2^−/−^ mice between d 20 and 60 pi could have helped determine whether there was a complete restoration of responsiveness in the absence of PD‐L2. Unfortunately, this data was not available.

## 5 Materials and Methods

### 5.1 Ethics Statement

All animal work was approved by the University of Edinburgh Ethics Committee (PL02‐10) and by the UK Home Office (PPL70/8548), and conducted in accordance with the Animals (Scientific Procedures) Act 1986.

### 5.2 Animals, Parasites, and Cell Isolations

Female BALB/c, PD‐L2^−/−^ [42], and Jh^−/−^ [43] mice on the BALB/c background were bred in‐house and maintained under specific pathogen‐free conditions at the University of Edinburgh. Mice were used at 6–12 weeks of age, and randomly assigned to experimental groups. The *L. sigmodontis* life cycle was maintained in gerbils (*Meriones unguiculatus*) using the mite vector *Ornithonyssus bacoti* [24, 44]. Mice were infected s.c. on the upper back with 30 *L. sigmodontis* L_3_ larvae. *L. sigmodontis* antigen was prepared by collecting the PBS‐soluble fraction of homogenized adult male and female parasites. To quantify blood microfilariae, 30 µL of tail blood was collected in FACSlysing solution (Becton‐Dickinson), and microfilariae were counted using dark field optical microscopy (Axiovert 25, Zeiss). The para‐thymic, posterior, mediastinal and paravertebral LN were taken as a source of thoracic LN (tLN) draining the pleural cavity (PleC). TLN cells were dissociated and washed in RPMI‐1640 (Invitrogen) supplemented with 0.5% mouse sera (Cal‐tag‐Medsystems), 100 U/mL penicillin, 100 µg/mL streptomycin and 2 mM L‐glutamine. PleC cells were recovered by lavage and washed as above.

### 5.3 Flow Cytometry and Intracellular Cytokine Staining

The following antibodies were used: Pacific Blue‐ or AlexaFluor 700‐conjugated anti‐CD4 (RM4‐5), phycoerythrin (PE)‐conjugated anti‐IL‐4 (11B11, Biolegend), allophycocyanine (APC)‐conjugated anti‐IL‐5 (TRFK5, Biolegend), AlexaFluor 700‐conjugated anti‐Foxp3 (FJK‐16s, eBioscience), biotinylated anti‐CXCR5 (2G8, BD Biosciences), Pe‐Cy7‐conjugated anti‐IFN‐γ (XMG1.2, Biolegend), FITC‐conjugated anti‐GATA3 (REA174, Miltenyi Biotech), PE‐conjugated anti‐PD‐L2 (Ty25, eBioscience), Pacific‐Blue‐conjugated anti‐IgD (11‐26, Sourthern Biotech), PE‐conjugated polyclonal goat anti‐mouse IgM (Southern Biotech), Pe‐Cy7‐conjugated anti‐CD19 (6D5, Biolegend), AlexaFluor 700‐conjugated anti‐Igκ (187.1, BD Biosciences), PerCP‐conjugated anti‐B220 (RA3‐6B2, Biolegend), APC‐conjugated anti‐CD138 (281‐2, Biolegend), APC‐conjugated Streptavidin (Biolegend), FITC‐conjugated peanut agglutinin (PNA) (Vector Laboratories). Nonspecific binding was blocked with 4 µg of rat IgG/1 × 10^6^ cells. Staining of transcription factors was performed using the Foxp3/Transcription Factor Staining Buffer Set (Ebioscience). For intracellular cytokine staining 2 × 10^6^ cells/well were stimulated in RPMI‐1640 media (Invitrogen), containing 100 U/mL Penicillin, 100 µg/mL Streptomycin, 2 mM l‐Glutamine, 0.5 µg/mL PMA and 1 µg/mL Ionomycin (both Sigma) at 37°C and 5% CO_2_ for 4 h. Brefeldin A (Invitrogen) was added at a final concentration of 10 µg/mL for the final 2 h. Dead cells were excluded by the Zombie Aqua Fixable Viability kit (Biolegend), and the cells were fixed and permeabilised using Biolegend fixation buffer in combination with the Intracellular Permeabilisation Wash Buffer. Flow cytometric acquisition was performed using a FACS Canto II (BD Biosciences), and data were analysed using Flowjo Software (Tree Star).

### 5.4 Bone Marrow Chimaeras

Bone marrow chimeric mice were generated as described previously [45]. Briefly, host B cell‐deficient Jh^−/−^ mice were irradiated with two doses of 5 Gy gamma‐radiation approximately 3–4 h apart. The following day, mice were reconstituted with 4 × 10^6^ mixed‐inoculum bone marrow cells in the following ratios: 80% Jh^−/−^ with 20% PD‐L2^−/−^, 80% WT with 20% PD‐L2^−/−^, and 80% Jh^−/−^ with 20% WT bone marrow. Infections were performed 9 weeks post‐bone marrow inoculation.

### 5.5 *L. sigmodontis*‐Specific Antibody ELISA

ELISA plates (NUNC) were coated with 5 µg/mL *L. sigmodontis* Ag diluted in 0.45 M NaHCO3/0.18MNa2CO3 (Sigma‐Aldrich). Plates were incubated with serial dilutions of serum, and a representative dilution from the linear section of the dilution curve was selected for statistical analysis and visualisation of results. Detection of IgG1 isotype was performed using HRP‐conjugated anti‐mouse IgG1 (Southern Biotechnology Associates) and ABTS per‐oxidase substrate system (KPL).

### 5.6 Statistical Analysis

Statistical analyses were performed in RStudio using R. Data were analysed using multi‐factor linear models (LM) or generalised linear models (GLM), followed by Tukey's post hoc tests as required. All models used combined data from multiple experiments with ‘experimental repeat’ included as an independent variable to account for baseline differences between independent repeats, and to ensure results were qualitatively similar between repeats. Models were initially run with all independent variables and all interaction terms, and simplified by stepwise removal of non‐significant terms starting with the most complex and then those with the highest *p*‐value. All starting and final models are listed in Table S1. To confirm the data fitted the assumptions of LM, the residuals were checked for normal distribution, and the residuals versus fitted values were checked for a linear relationship.

## Author Contributions

**Matthew Taylor**: Conceptualisation, formal analysis, funding acquisition, investigation, methodology, supervision, writing — original draft, writing — review and editing. **Johanna Knipper**: Conceptualisation, data curation, formal analysis, investigation, methodology, writing — original draft, review and editing. **Sharon Campbell**: Data curation, investigation, conceptualisation, writing — review and editing. **Judith Allen**: Conceptualisation, funding acquisition, writing — review and editing.

## Conflicts of Interest

The authors declare no conflicts of interest.

## Peer Review

The peer review history for this article is available at https://publons.com/publon/10.1002/eji.70021.

## Supporting information




**Supporting File 1**: eji70021‐sup‐0001‐SuppMat.pdf.

**Supporting File 2**: eji70021‐sup‐0002‐TableS2.xlsx.

## Data Availability

Data are available in the article's Supporting Information Material (Table S2).
