# Supplementary material for: PD‐L2 Inhibits Protective Immunity, Th2 Cell Functional Quality, and GATA‐3 Expression During Filarial Nematode Infection
Source: Eur J Immunol. 2025 Aug 4;55(8):e70021. doi: 10.1002/eji.70021 (PMC12319375; doi:10.1002/eji.70021)
Supplement: Supplementary file 1 — Supporting File 1: eji70021‐sup‐0001‐SuppMat.pdf. [file EJI-55-e70021-s001.pdf]

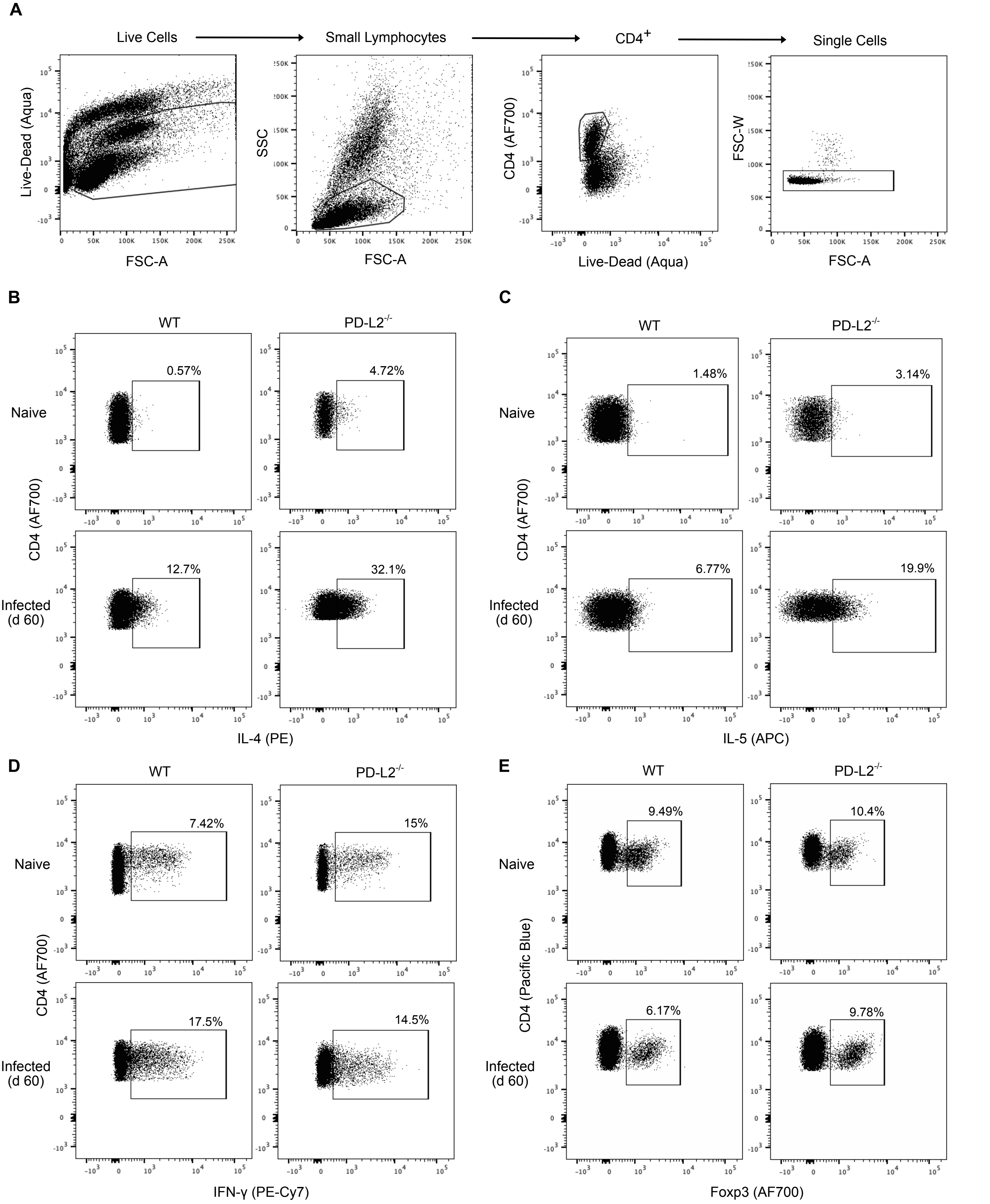

Supplemental Figure 1. Representative FC gating approaches for cytokine production and Foxp3 expression by CD4 T cells. (A) Gating approach to identify live $CD4^+$ T cells. (B – D) Representative FC staining showing IL-4 (B), IL-5 (C), and IFN-γ (D) production by PleC $CD4^+$ T cells in naïve and infected WT and PD-$L2^{-/-}$ mice at d 60 pi. (E) Representative FC staining showing Foxp3 expression by PleC $CD4^+$ T cells in naïve and infected WT and PD-$L2^{-/-}$ mice at d 60 pi.

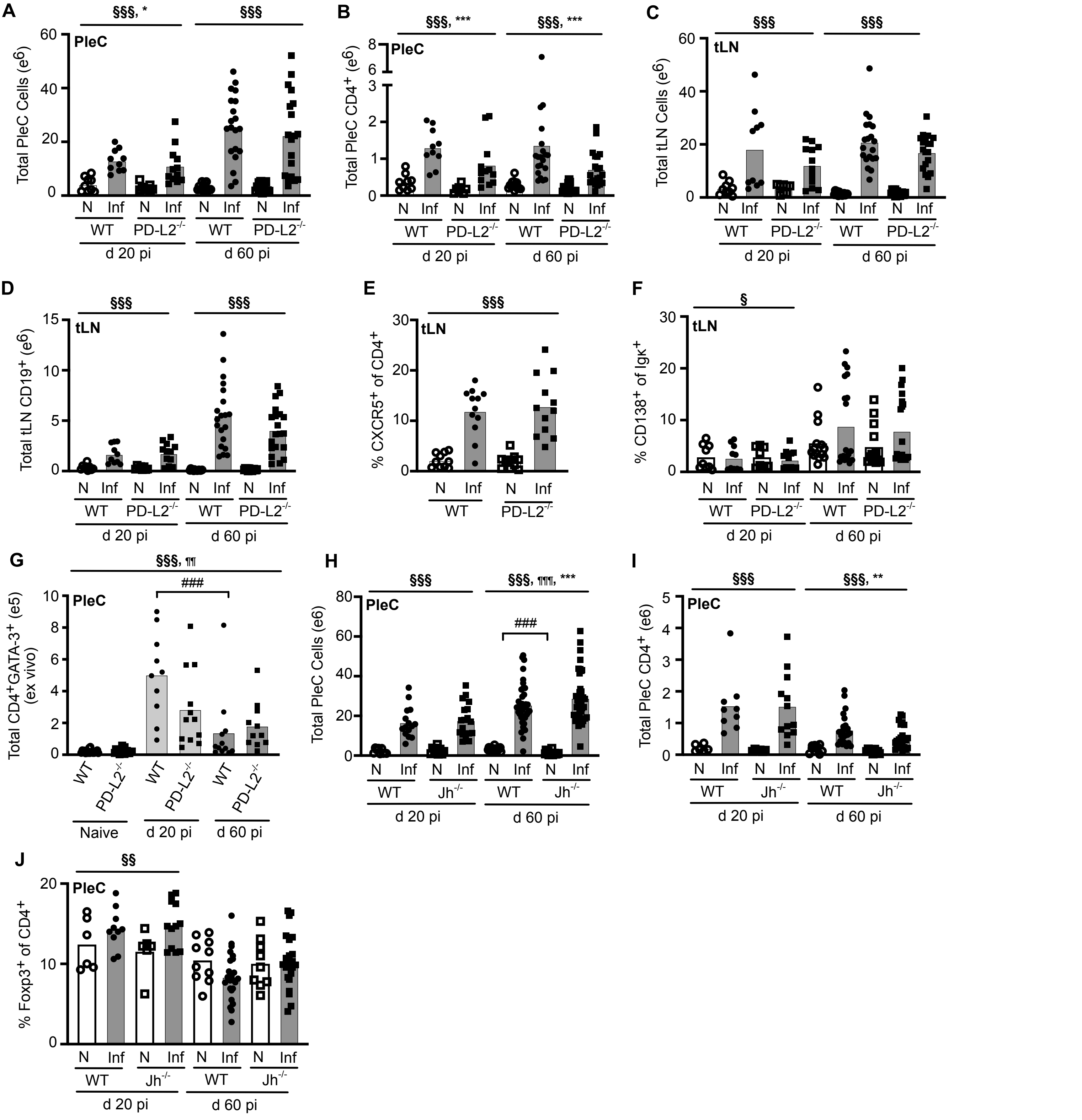

Supplemental Figure 2. Changes in immune cell populations in PD-L2$^{-/-}$ and Jh$^{-/-}$ mice. Symbols represent individual mice and bars represent the mean. (A – G) Immune cell populations were assessed in naïve (N, open symbols) and *L. sigmodontis*-infected (Inf, closed symbols) WT (circles) and PD-L2$^{-/-}$ (squares) mice at d 20 and 60 pi. Total number of PleC cells (A), PleC CD4^{+} T cells (B), tLN cells (C), and tLN CD19^{+} B cells (D). Percentage of CD4^{+} T cells expressing CXCR5 at d 60 pi (E), Igκ^{+} B cells expressing CD138 at d 20 and 60 pi (F). Total numbers of PleC CD4^{+}GATA-3^{+} T cells (G). (A – D, & F) Data from 2 (d 20) or 3 (d 60) independent experiments: N WT (d20 n=9, d60 n=13), Inf WT (d20 n=10, d60 n=20), N PD-L2$^{-/-}$ (d20 n=8, d60 n = 13), Inf PD-L2$^{-/-}$ (d20 n=12, d60 n=19). (E) Data from 2 independent experiments: N WT (n=10) Inf WT (n=12), N PD-L2$^{-/-}$ (n=10), Inf PD-L2$^{-/-}$ (n=11). (G) Data from 2 independent experiments; N WT (n=19), N PD-L2$^{-/-}$ (n=18), infected WT (d20 n=9-10, d 60 n=12), infected PD-L2$^{-/-}$ (d20 n=12, d60 n=11-12). Naïve animals were pooled across timepoints and data analysed using LM. (H – J) Immune cell populations were assessed in naïve (N, open symbols) and *L. sigmodontis*-infected (Inf, closed symbols) WT (circles) and Jh$^{-/-}$ (squares) mice at d 20 and 60 pi. Total number of PleC cells (H) and PleC CD4^{+} T cells (I), and percentage of CD4^{+} T cells expressing Foxp3 (J). (H) Data from 3 (d20) or 5 (d60) independent experiments: N WT (d20 n=9, d60 n=18), Inf WT (d20 n=15, d60 n=37), N Jh$^{-/-}$ (d20 n=9, d60 n=9), Inf Jh$^{-/-}$ (d20 n=18, d60 n=38). (I & J) Data from 2 (d20) or 3 (d60) independent experiments: N WT (d20 n=6, d60 n=10), Inf WT (d20 n=9-10, d60 n=24), N Jh$^{-/-}$ (d20 n=6, d60 n=9-10), Inf Jh$^{-/-}$ (d20 n=12, d60 n=24). (A – J) Significant effect of infection at $^{§§§}p < 0.001$, $^{§§}p < 0.01$ or $^{§}p < 0.05$, significant effect of genotype at $^{***}p < 0.001$, $^{**}p < 0.01$ or $^{*}p < 0.05$, significant effect of genotype*infection at $^{¶¶¶}p < 0.001$ or $^{¶¶}p < 0.01$ (LM). $^{###}p < 0.001$ (Tukeys HSD).

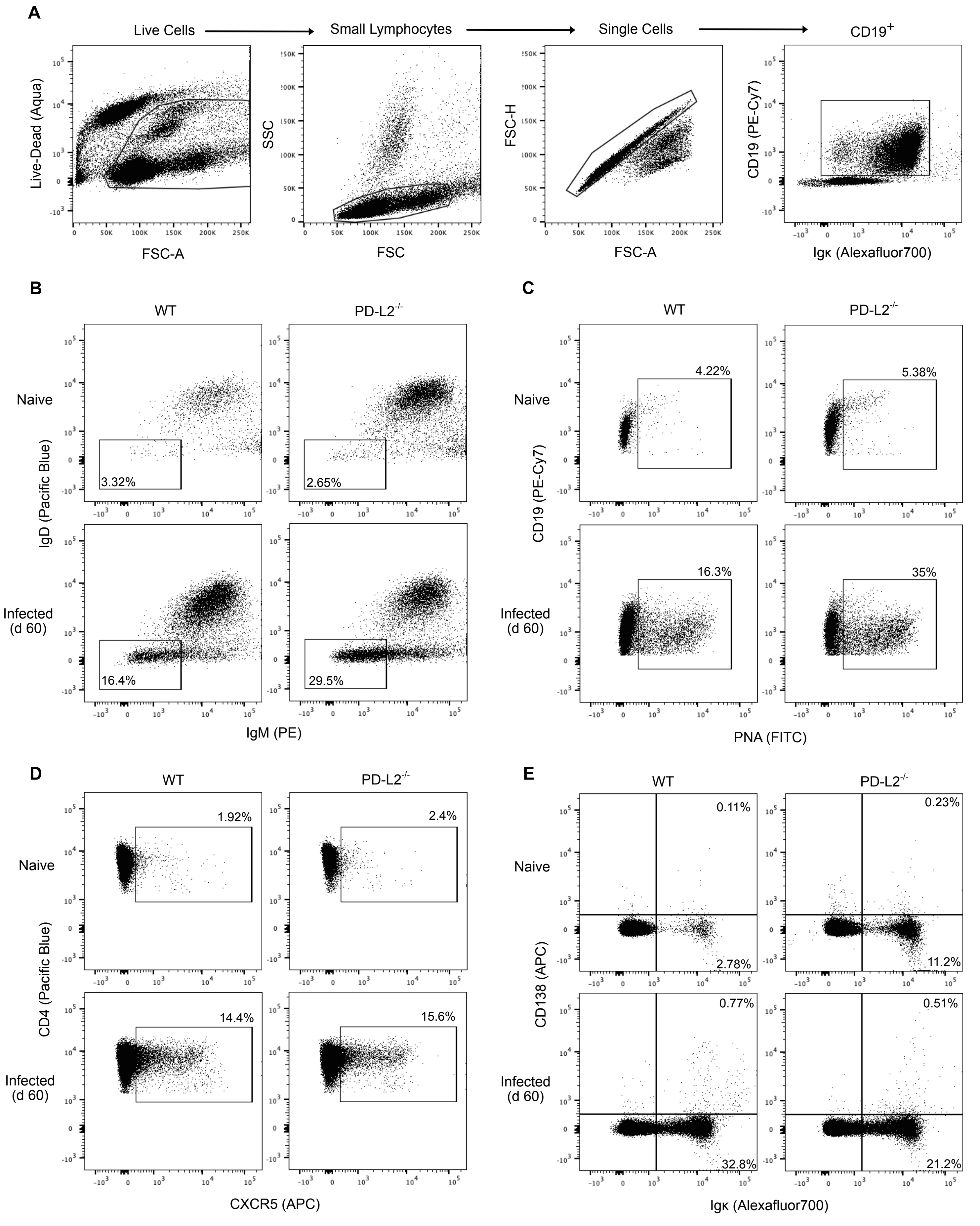

Supplemental Figure 3. Representative FC gating approaches for B cells and CD4⁺ Tfh cells in naïve and *L. sigmodontis*-infected WT and PD-L2$^{-/-}$ mice. (A) Gating approach to identify live CD19$^+$ T cells. (B - E) Representative staining for IgM$^-$IgD$^-$ class switched CD19$^+$ B cells (B), CD19$^+$PNA$^+$ germinal centre B cells (C), Igκ$^+$ CD138$^+$ plasma cells (D), and CD4$^+$CXC5$^+$ Tfh cells (E).

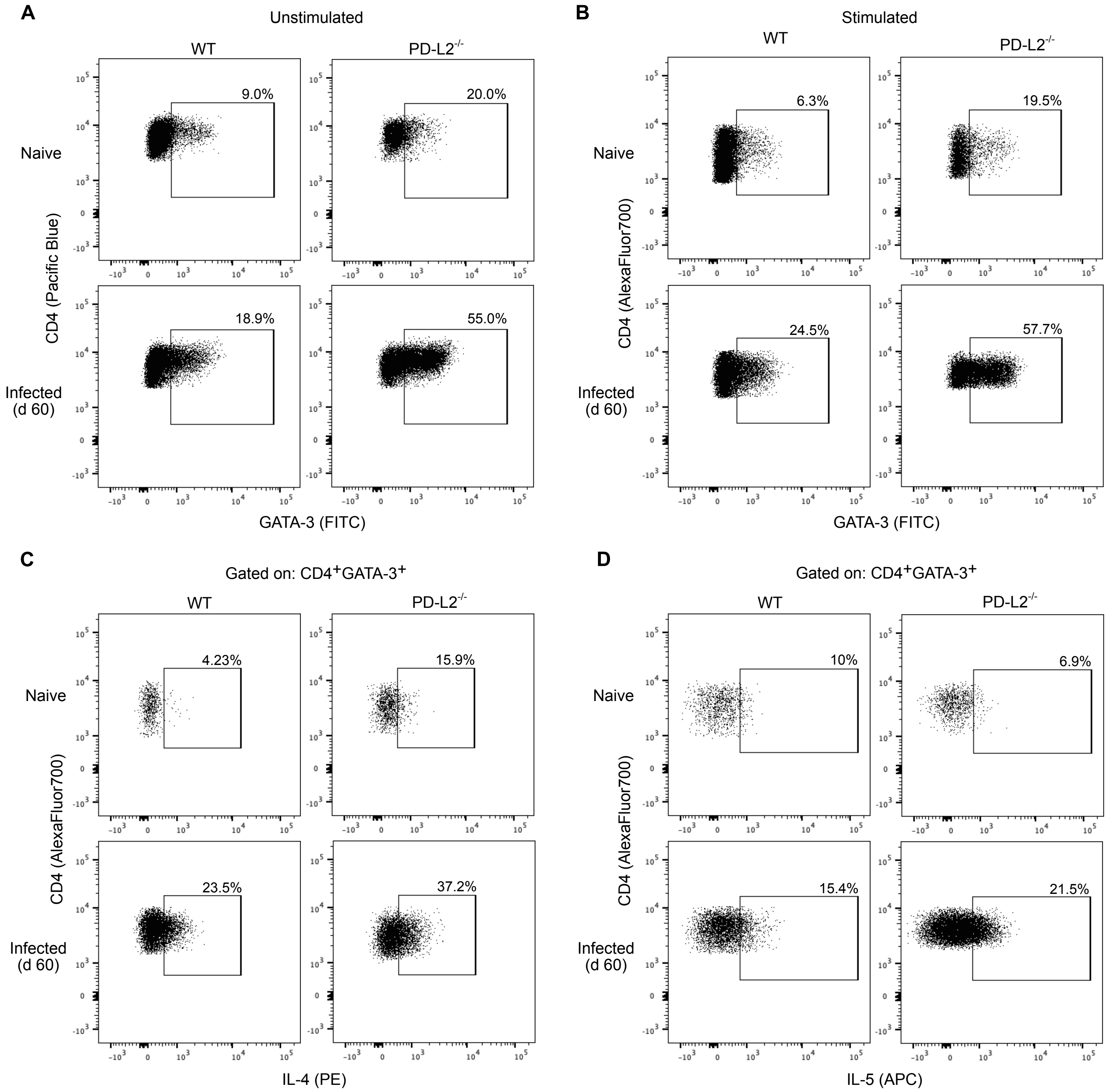

Supplemental Figure 4. Representative FC gating approaches for GATA-3 expression by CD4⁺ T cells and cytokine production by CD4⁺GATA-3⁺ T cells in naïve and *L. sigmodontis*-infected WT and PD-L2$^{-/-}$ mice. (A – D) Representative staining for GATA-3 expression by unstimulated CD4⁺ T cells (A), GATA-3 expression by stimulated CD4⁺GATA-3⁺ T cells (B), IL-4 production by CD4⁺GATA-3⁺ T cells (C), and IL-5 production by CD4⁺GATA-3⁺ T cells (D).

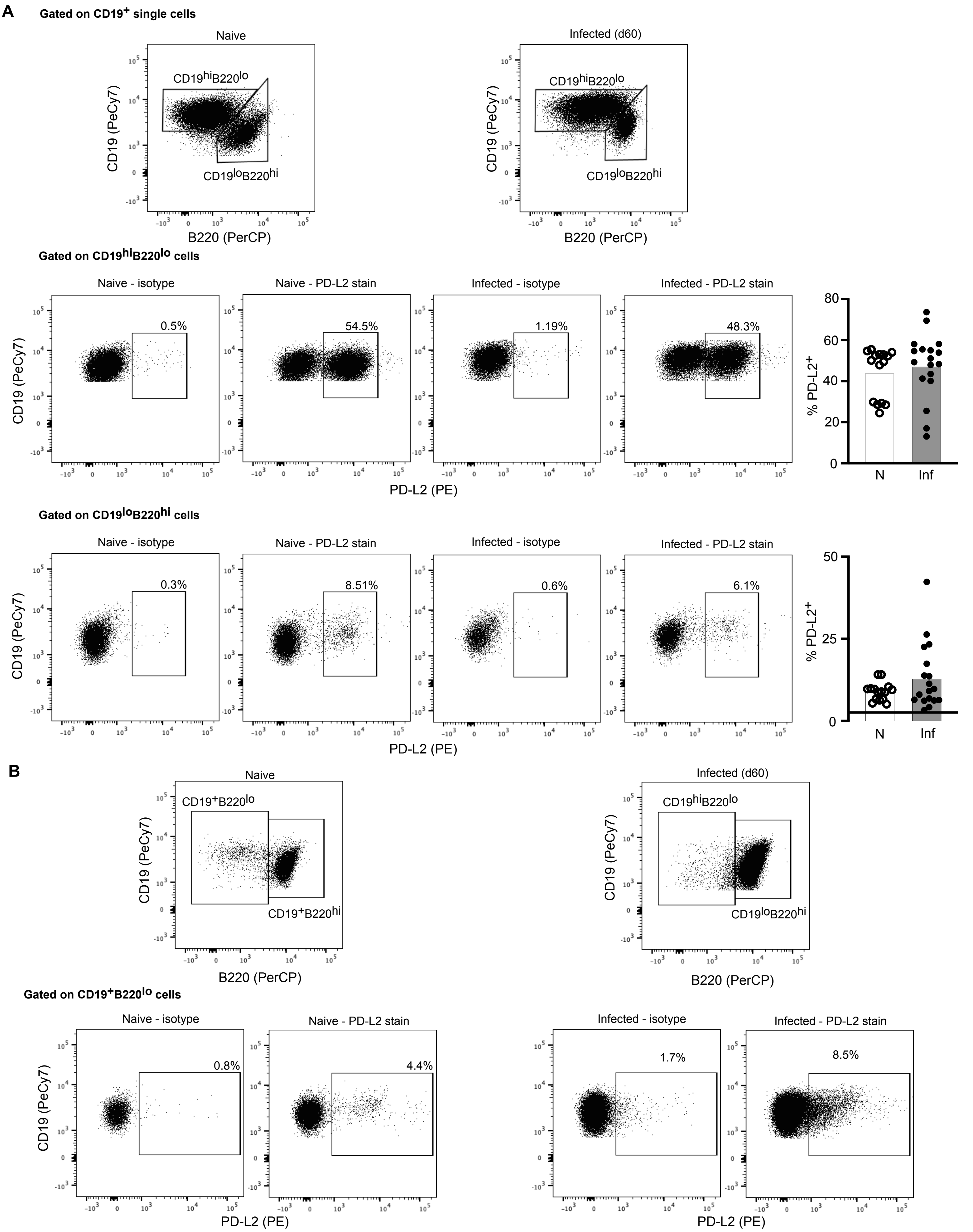

Supplemental Figure 5. Representative FC gating approaches to measure PD-L2 expression on CD19⁺ B cells. (A) Flow plots show representative staining of PD-L2 on PleC CD19^{hi}B220^{lo} and CD19^{lo}B220^{hi} B cell populations from naïve and *L. sigmodontis* infected WT mice 60 d pi. Graphs show percentage of PleC CD19^{hi}B220^{lo} and CD19^{lo}B220^{hi} B cells expressing PD-L2. Open and closed symbols represent individual naïve (N) and infected (Inf) mice respectively, bars represent the mean. Data from 3 independent experiments: N (n=15), Inf (n=18). (B) Representative staining of PD-L2 on tLN CD19^{+}B220^{lo} and CD19^{+}B220^{hi} B cell populations from naïve and *L. sigmodontis* infected WT mice 60 d pi.

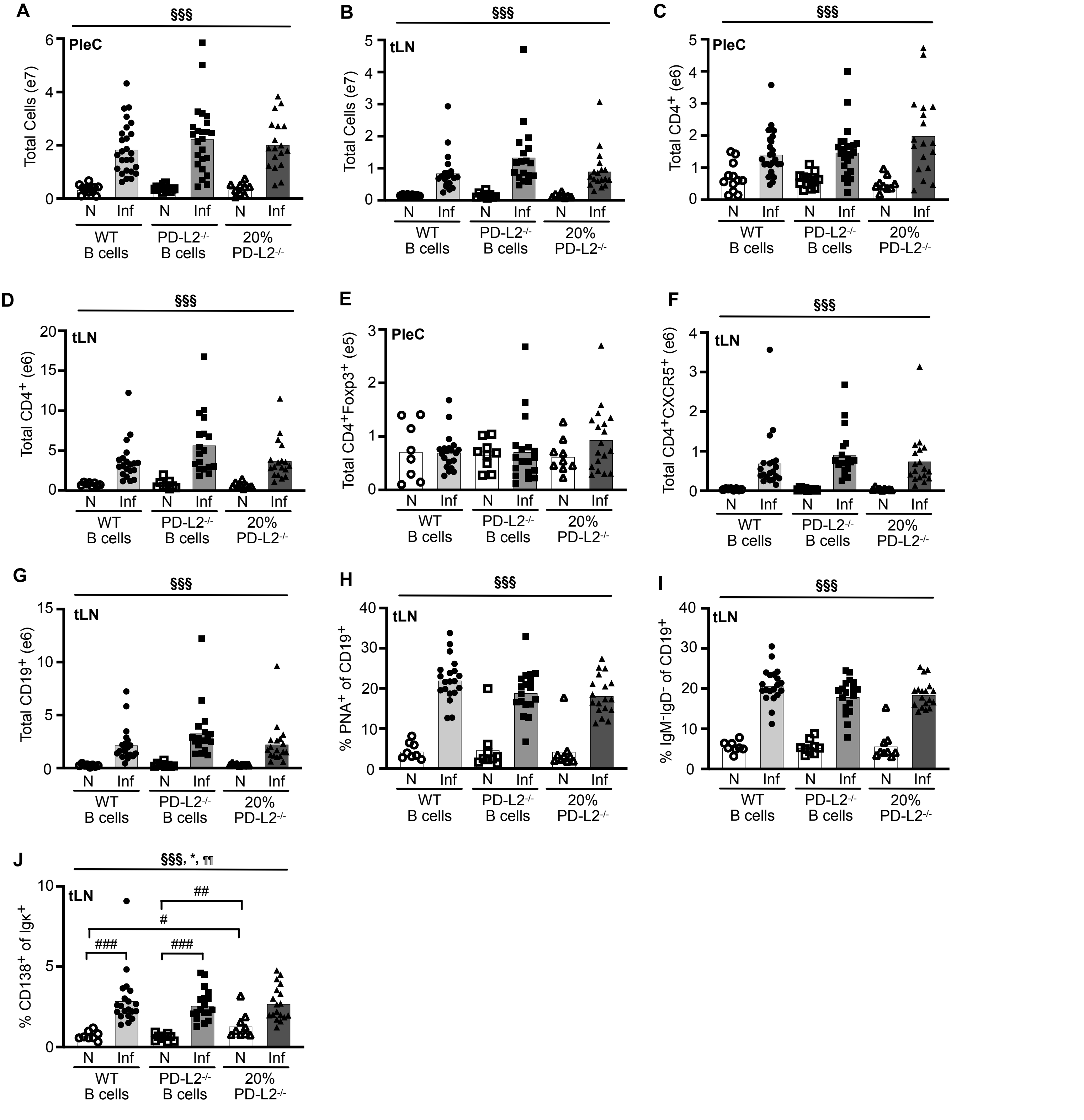

Supplemental Figure 6. Phenotyping of bone marrow chimaeras. B cell expression of PD-L2 and changes in immune cell populations were assessed in naïve (N, open symbols)) and d 60 *L. sigmodontis*-infected (Inf, closed symbols) B cell chimaeras that contained B cells deficient for PD-L2 (squares), or control chimaeras generated with WT B cells (circles) or 80%/20% WT/PD-L2$^{-/-}$ (triangles) bone marrow. Symbols represent individual mice and bars represent the mean. (A - G) Total number of PleC cells (A), tLN cells (B), PleC CD4$^+$ T cells (C), tLN CD4$^+$ T cells (D), PleC CD4$^+$Foxp3$^+$ T cells (E), tLN CD4$^+$CXCR5$^+$ Tfh cells (F), and tLN CD19$^+$ B cells (G). (H – J) Percentage of CD19$^+$PNA$^+$ GC B cells (H), CD19$^+$IgM$^-$IgD$^-$ class-switched B cells (I), and Igκ$^+$CD138$^+$ plasma cells (J). (A, C) Data from 3-independent experiments: N WT B cell (n=12), Inf WT B cell (n=26), N B cell PD-L2$^{-/-}$ (n=13), Inf B cell PD-L2$^{-/-}$ (n=24), N 20% PD-L2$^{-/-}$ (n=9), Inf 20% PD-L2$^{-/-}$ (n=18). (B, D – J) Data from 2-independent experiments: N WT B cell (n=8-9), Inf WT B cell (n=20), N B cell PD-L2$^{-/-}$ (n=9), Inf B cell PD-L2$^{-/-}$ (n=18), N 20% PD-L2$^{-/-}$ (n=8-9), Inf 20% PD-L2$^{-/-}$ (n=18). (A – J) §§§Significant effect of infection at $p < 0.001$, *significant effect of group at $p < 0.05$, ¶¶significant effect of chimaera group*infection at $p < 0.01$ (LM). $^{###}p < 0.001$, $^{##}p < 0.01$ $^{\#}p < 0.05$ (Tukey's HSD).

Supplemental Table 1: Starting and final statistical models for each figure.

**Figure 1A**	
Starting	*Experiment + Genotype + Timepoint + Experiment*Genotype + Experiment*Timepoint + Genotype*Timepoint + Experiment*Genotype*Timepoint*
Final	Genotype + Timepoint
**Figure 1B**	
Starting	*Experiment + Genotype + Timepoint + Experiment*Genotype*
Final	Experiment + Genotype
**Figure 1C**	
Starting d20	*Experiment + Infection + Genotype + Experiment*Infection + Experiment*Genotype + Genotype*Infection + Experiment*Infection*Genotype*
Final d20	Infection + Genotype + Infection*genotype
Starting d60	*Experiment + Infection + Genotype + Experiment*Infection + Experiment*Genotype + Genotype*Infection + Experiment*Infection*Genotype*
Final d60	Experiment + Infection + Genotype + Infection*genotype
**Figure 1D**	
Starting d20	*Experiment + Infection + Genotype + Experiment*Infection + Experiment*Genotype + Genotype*Infection + Experiment*Infection*Genotype*
Final d20	Infection + Genotype + Infection*genotype
Starting d60	*Experiment + Infection + Genotype + Experiment*Infection + Experiment*Genotype + Genotype*Infection + Experiment*Infection*Genotype*
Final d60	Experiment + Infection + Genotype + Experiment*Genotype + Infection*genotype
**Figure 1E**	
Starting d20	*Experiment + Infection + Genotype + Experiment*Infection + Experiment*Genotype + Genotype*Infection + Experiment*Infection*Genotype*
Final d20	Infection + Genotype + Infection*genotype
Starting d60	*Experiment + Infection + Genotype + Experiment*Infection + Experiment*Genotype + Genotype*Infection + Experiment*Infection*Genotype*
Final d60	Experiment + Infection + Genotype + Experiment*Infection + Experiment*Genotype + Infection*genotype
**Figure 1F**	
Starting	*Experiment + Genotype + Experiment*Genotype*
Final	Genotype
**Figure 1G**	
Starting d20	*Experiment + Infection + Genotype + Experiment*Infection + Experiment*Genotype + Genotype*Infection + Experiment*Infection*Genotype*
Final d20	Experiment + Infection + Genotype + Experiment*Infection + Experiment*Genotype + Genotype*Infection + Experiment*Infection*Genotype
Starting d60	*Experiment + Infection + Genotype + Experiment*Infection + Experiment*Genotype + Genotype*Infection + Experiment*Infection*Genotype*
Final d60	Experiment + Infection + Genotype + Infection*genotype
**Figure 1H**	
Starting d20	*Experiment + Infection + Genotype + Experiment*Infection + Experiment*Genotype + Genotype*Infection + Experiment*Infection*Genotype*
Final d20	Experiment + Infection + Genotype + Experiment*Infection + Experiment*Genotype + Genotype*Infection + Experiment*Infection*Genotype
Starting d60	*Experiment + Infection + Genotype + Experiment*Infection + Experiment*Genotype + Genotype*Infection + Experiment*Infection*Genotype*
Final d60	Experiment + Infection + Genotype + Experiment*Infection + Experiment*Genotype + Genotype*Infection + Experiment*Infection*Genotype
**Figure 1I**	

Starting	*Experiment + Infection + Genotype + Experiment*Infection + Experiment*Genotype + Genotype*Infection + Experiment*Infection*Genotype*
Final	Experiment + Infection + Genotype + Experiment*Infection + Experiment*Genotype + Genotype*Infection + Experiment*Infection*Genotype
**Figure 2A**	
Starting	*Experiment + timepoint + Genotype + Experiment*timepoint + Experiment*Genotype + Genotype*timepoint + Experiment*timepoint*Genotype*
Final	Experiment + timepoint + Genotype + Genotype*timepoint
**Figure 2B**	
Starting	*Experiment + timepoint + Genotype + Experiment*timepoint + Experiment*Genotype + Genotype*timepoint + Experiment*timepoint*Genotype*
Final	Experiment + timepoint + Genotype + Genotype*timepoint
**Figure 2C**	
Starting	*Experiment + Infection + Genotype + Experiment*Infection + Experiment*Genotype + Genotype*Infection + Experiment*Infection*Genotype*
Final	Experiment + Infection + Genotype + Genotype*Infection
**Figure 2D**	
Starting	*Experiment + Infection + Genotype + Experiment*Infection + Experiment*Genotype + Genotype*Infection + Experiment*Infection*Genotype*
Final	Experiment + Infection + Genotype + Genotype*Infection
**Figure 2E**	
Starting	*Experiment + Infection + Genotype + Experiment*Infection + Experiment*Genotype + Genotype*Infection + Experiment*Infection*Genotype*
Final	Experiment + Infection + Genotype + Genotype*Infection
**Figure 2F**	
Starting	*Experiment + Infection + Genotype + Experiment*Infection + Experiment*Genotype + Genotype*Infection + Experiment*Infection*Genotype*
Final	Experiment + Infection + Genotype + Genotype*Infection
**Figure 3A**	
Starting	*Experiment + Infection + Experiment*Infection*
Final	Experiment + Infection
**Figure 3B**	
Starting d20	*Experiment + Genotype + Experiment*Genotype*
Final d20	Experiment + Genotype
Starting d60	*Experiment + Genotype + Experiment*Genotype*
Final d60	Experiment + Genotype
**Figure 3C**	
Starting	*Experiment + Genotype + Experiment*Genotype*
Final	Experiment + Genotype
**Figure 3D**	
Starting	*Experiment + Timepoint + Infection + Genotype + Experiment*Timepoint + Experiment*Infection + Experiment*Genotype + Timepoint*Infection + Timepoint*Genotype + Infection*Genotype + Experiment*Timepoint*Infection + Experiment*Timepoint*Genotype + Experiment* Infection*Genotype + Timepoint*Infection*Genotype + Experiment*Timepoint* Infection*Genotype*
Final	Experiment + Timepoint + Infection + Genotype + Experiment*Timepoint + Experiment*Infection + Experiment*Genotype + Timepoint*Infection + Infection*Genotype + Experiment* Infection*Genotype
**Figure 3E**	
Starting	*Experiment + Timepoint + Infection + Genotype + Experiment*Timepoint + Experiment*Infection + Experiment*Genotype + Timepoint*Infection + Timepoint*Genotype + Infection*Genotype + Experiment*Timepoint*Infection +*

	*Experiment*Timepoint*Genotype + Experiment* Infection*Genotype + Timepoint*Infection*Genotype + Experiment*Timepoint* Infection*Genotype*
Final	Experiment + Timepoint + Infection + Genotype + Infection*Genotype
**Figure 3F**	
Starting	*Experiment + Timepoint + Infection + Genotype + Experiment*Timepoint + Experiment*Infection + Experiment*Genotype + Timepoint*Infection + Timepoint*Genotype + Infection*Genotype + Experiment*Timepoint*Infection + Experiment*Timepoint*Genotype + Experiment* Infection*Genotype + Timepoint*Infection*Genotype + Experiment*Timepoint* Infection*Genotype*
Final	Experiment + Timepoint + Infection + Genotype + Timepoint*Infection + Timepoint*Genotype + Infection*Genotype + Timepoint*Infection*Genotype
**Figure 3G**	
Starting	*Experiment + Timepoint + Infection + Genotype + Experiment*Timepoint + Experiment*Infection + Experiment*Genotype + Timepoint*Infection + Timepoint*Genotype + Infection*Genotype + Experiment*Timepoint*Infection + Experiment*Timepoint*Genotype + Experiment* Infection*Genotype + Timepoint*Infection*Genotype + Experiment*Timepoint* Infection*Genotype*
Final	Experiment + Timepoint + Infection + Genotype + Timepoint*Genotype + Infection*Genotype
**Figure 3H**	
Starting	*Experiment + Timepoint + Infection + Genotype + Experiment*Timepoint + Experiment*Infection + Experiment*Genotype + Timepoint*Infection + Timepoint*Genotype + Infection*Genotype + Experiment*Timepoint*Infection + Experiment*Timepoint*Genotype + Experiment* Infection*Genotype + Timepoint*Infection*Genotype + Experiment*Timepoint* Infection*Genotype*
Final	Experiment + Timepoint + Infection + Genotype + Experiment*Timepoint + Experiment*Infection + Experiment*Genotype + Timepoint*Infection + Infection*Genotype + Experiment* Infection*Genotype
**Figure 4B**	
Starting	*Experiment + Group + Experiment*Group*
Final	Group
**Figure 4C**	
Starting	*Experiment + Group + Experiment*Group*
Final	Experiment + Group
**Figure 4D**	
Starting	*Experiment + Infection + Group + Experiment*Infection + Experiment*Group + Infection*Group + Experiment*Infection*Group*
Final	Experiment + Infection + Group
**Figure 4E**	
Starting	*Experiment + Infection + Group + Experiment*Infection + Experiment*Group + Infection*Group + Experiment*Infection*Group*
Final	Experiment + Infection + Group + Experiment*Infection
**Figure 4F**	
Starting	*Experiment + Infection + Group + Experiment*Infection + Experiment*Group + Infection*Group + Experiment*Infection*Group*
Final	Experiment + Infection + Group
**Figure 4G**	
Starting	*Experiment + Infection + Group + Experiment*Infection + Experiment*Group + Infection*Group + Experiment*Infection*Group*
Final	Experiment + Infection + Group + Experiment*Infection
**Figure 4H**	
Starting	*Experiment + Infection + Group + Experiment*Infection + Experiment*Group + Infection*Group + Experiment*Infection*Group*

Final	Infection + Group
**Figure S2A**	
Starting d20	*Experiment + Infection + Genotype + Experiment*Infection + Experiment*Genotype + Genotype*Infection + Experiment*Infection*Genotype*
Final d20	Infection + Genotype + Experiment*Infection + Experiment*Genotype
Starting d60	*Experiment + Infection + Genotype + Experiment*Infection + Experiment*Genotype + Genotype*Infection + Experiment*Infection*Genotype*
Final d60	Experiment + Infection + Genotype + Experiment*Infection
**Figure S2B**	
Starting d20	*Experiment + Infection + Genotype + Experiment*Infection + Experiment*Genotype + Genotype*Infection + Experiment*Infection*Genotype*
Final d20	Infection + Genotype + Experiment*Infection + Experiment*Genotype
Starting d60	*Experiment + Infection + Genotype + Experiment*Infection + Experiment*Genotype + Genotype*Infection + Experiment*Infection*Genotype*
Final d60	Experiment + Infection + Genotype + Experiment*Infection
**Figure S2C**	
Starting d20	*Experiment + Infection + Genotype + Experiment*Infection + Experiment*Genotype + Genotype*Infection + Experiment*Infection*Genotype*
Final d20	Experiment + Infection + Genotype + Experiment*Infection + Experiment*Genotype + Genotype*Infection + Experiment*Infection*Genotype
Starting d60	*Experiment + Infection + Genotype + Experiment*Infection + Experiment*Genotype + Genotype*Infection + Experiment*Infection*Genotype*
Final d60	Experiment + Infection + Genotype + Experiment*Infection
**Figure S2D**	
Starting d20	*Experiment + Infection + Genotype + Experiment*Infection + Experiment*Genotype + Genotype*Infection + Experiment*Infection*Genotype*
Final d20	Experiment + Infection + Genotype + Experiment*Infection + Experiment*Genotype + Genotype*Infection + Experiment*Infection*Genotype
Starting d60	*Experiment + Infection + Genotype + Experiment*Infection + Experiment*Genotype + Genotype*Infection + Experiment*Infection*Genotype*
Final d60	Experiment + Infection + Genotype + Experiment*Infection + Experiment*Genotype + Genotype*Infection + Experiment*Infection*Genotype
**Figure S2E**	
Starting	*Experiment + Infection + Genotype + Experiment*Infection + Experiment*Genotype + Genotype*Infection + Experiment*Infection*Genotype*
Final	Experiment + Infection + Genotype
**Figure S2F**	
Starting d20	*Experiment + Infection + Genotype + Experiment*Infection + Experiment*Genotype + Genotype*Infection + Experiment*Infection*Genotype*
Final d20	Experiment + Infection + Genotype + Experiment*Infection + Experiment*Genotype + Genotype*Infection + Experiment*Infection*Genotype
Starting d60	*Experiment + Infection + Genotype + Experiment*Infection + Experiment*Genotype + Genotype*Infection + Experiment*Infection*Genotype*
Final d60	Experiment + Infection + Genotype + Experiment*Infection + Experiment*Genotype + Genotype*Infection + Experiment*Infection*Genotype
**Figure S2G**	
Starting	*Experiment + Infection + Genotype + Experiment*Infection + Experiment*Genotype + Genotype*Infection + Experiment*Infection*Genotype*
Final	Experiment + timepoint + Genotype + Genotype*timepoint
**Figure S2H**	
Starting d20	*Experiment + Infection + Genotype + Experiment*Infection + Experiment*Genotype + Genotype*Infection + Experiment*Infection*Genotype*

Final d20	Experiment + Infection + Genotype + Experiment*Infection + Experiment*Genotype + Genotype*Infection + Experiment*Infection*Genotype
Starting d60	*Experiment + Infection + Genotype + Experiment*Infection + Experiment*Genotype + Genotype*Infection + Experiment*Infection*Genotype*
Final d60	Experiment + Infection + Genotype + Experiment*Infection + Experiment*Genotype + Genotype*Infection + Experiment*Infection*Genotype
**Figure S2I**	
Starting d20	*Experiment + Infection + Genotype + Experiment*Infection + Experiment*Genotype + Genotype*Infection + Experiment*Infection*Genotype*
Final d20	Experiment + Infection + Genotype + Experiment*Infection
Starting d60	*Experiment + Infection + Genotype + Experiment*Infection + Experiment*Genotype + Genotype*Infection + Experiment*Infection*Genotype*
Final d60	Experiment + Infection + Genotype + Experiment*Infection + Experiment*Genotype + Genotype*Infection
**Figure S2J**	
Starting d20	*Experiment + Infection + Genotype + Experiment*Infection + Experiment*Genotype + Genotype*Infection + Experiment*Infection*Genotype*
Final d20	Experiment + Infection + Genotype + Experiment*Infection + Experiment*Genotype + Genotype*Infection + Experiment*Infection*Genotype
Starting d60	*Experiment + Infection + Genotype + Experiment*Infection + Experiment*Genotype + Genotype*Infection + Experiment*Infection*Genotype*
Final d60	Experiment + Infection + Genotype + Experiment*Infection + Experiment*Genotype + Genotype*Infection + Experiment*Infection*Genotype
**Figure S5A**	
Starting	*Experiment + Infection + Experiment*Infection*
Final	Experiment + Infection
**Figure S6A**	
Starting	*Experiment + Infection + Group + Experiment*Infection + Experiment*Group + Infection*Group + Experiment*Infection*Group*
Final	Experiment + Infection + Group
**Figure S6B**	
Starting	*Experiment + Infection + Group + Experiment*Infection + Experiment*Group + Infection*Group + Experiment*Infection*Group*
Final	Experiment + Infection + Group + Infection*Group
**Figure S6C**	
Starting	*Experiment + Infection + Group + Experiment*Infection + Experiment*Group + Infection*Group + Experiment*Infection*Group*
Final	Experiment + Infection + Group
**Figure S6D**	
Starting	*Experiment + Infection + Group + Experiment*Infection + Experiment*Group + Infection*Group + Experiment*Infection*Group*
Final	Infection + Group
**Figure S6E**	
Starting	*Experiment + Infection + Group + Experiment*Infection + Experiment*Group + Infection*Group + Experiment*Infection*Group*
Final	Experiment + Infection + Group + Experiment*Infection + Experiment*Group + Infection*Group + Experiment*Infection*Group
**Figure S6F**	
Starting	*Experiment + Infection + Group + Experiment*Infection + Experiment*Group + Infection*Group + Experiment*Infection*Group*
Final	Experiment + Infection + Group + Experiment*Infection + Experiment*Group + Infection*Group + Experiment*Infection*Group
**Figure S6G**	

Starting	*Experiment + Infection + Group + Experiment*Infection + Experiment*Group + Infection*Group + Experiment*Infection*Group*
Final	Experiment + Infection + Group
**Figure S6H**	
Starting	*Experiment + Infection + Group + Experiment*Infection + Experiment*Group + Infection*Group + Experiment*Infection*Group*
Final	Experiment + Infection + Group + Experiment*Group
**Figure S6I**	
a	*Experiment + Infection + Group + Experiment*Infection + Experiment*Group + Infection*Group + Experiment*Infection*Group*
Final	Experiment + Infection + Group
**Figure S6J**	
Starting	*Experiment + Infection + Group + Experiment*Infection + Experiment*Group + Infection*Group + Experiment*Infection*Group*
Final	Experiment + Infection + Group + Infection*Group
